# Oligomeric Interfaces Under the Lens: Gemini

**DOI:** 10.1371/journal.pone.0009897

**Published:** 2010-03-25

**Authors:** Giovanni Feverati, Claire Lesieur

**Affiliations:** Laboratoire de physique théorique LAPTH, CNRS, UMR 5108 associé à l'Université de Savoie, BP 110, Annecy le Vieux, France; Massachusetts Institute of Technology, United States of America

## Abstract

The assembly of subunits in protein oligomers is an important topic to study as a vast number of proteins exists as stable or transient oligomer and because it is a mechanism used by some protein oligomers for killing cells (e.g., perforin from the human immune system, pore-forming toxins from bacteria, phage, amoeba, protein misfolding diseases, etc.). Only a few of the amino acids that constitute a protein oligomer seem to regulate the capacity of the protein to assemble (to form interfaces), and some of these amino acids are localized at the interfaces that link the different chains. The identification of the residues of these interfaces is rather difficult. We have developed a series of programs, under the common name of Gemini, that can select the subset of the residues that is involved in the interfaces of a protein oligomer of known atomic structure, and generate a 2D interaction network (or graph) of the subset. The graphs generated for several oligomers demonstrate the accuracy of the selection of subsets that are involved in the geometrical and the chemical properties of interfaces. The results of the Gemini programs are in good agreement with those of similar programs with an advantage that Gemini programs can perform the residue selection much more rapidly. Moreover, Gemini programs can also perform on a single protein oligomer without the need of comparison partners. The graphs are extremely useful for comparative studies that would help in addressing questions not only on the sequence specificity of protein interfaces but also on the mechanism of the assembly of unrelated protein oligomers.

## Introduction

In cells, a vast majority of proteins function as oligomers which need to fold and assemble several copies of their chain (association step) to form a functional state [1]. These protein oligomers either oligomerize post-translationally or exist first in a monomeric state and oligomerize later in their life span. The latter is a key event in protein misfolding diseases such as Alzheimer. In this type of diseases, a protein (e.g. amyloid precursor) is initially produced as a non lethal monomer which after triggering, oligomerizes, destroys cells and induces the onset of the disease [2]. Similarly bacteria, viruses and several parasites (trypanosome, amoeba) also produce protein oligomers as the main virulence factors (e.g. cholera toxin, anthrax) [3].

What distinguishes protein oligomers from protein monomers is their unique capacity to self-assemble through the formation of interfaces (inter-subunit domains) between polypeptide chains. An interface is made by the association of one domain, provided by one chain, with another, provided by another chain. The domains that compose the interfaces are referred to as *segments* in the paper.

The following three aspects are important in understanding protein oligomers: (i) identification of the features of the interface (chemical and geometrical specificities), (ii) establishment of the assembly mechanism and (iii) determination of the molecular elements (e.g. amino acids) responsible for the mechanisms of assembly, and in particular for the association of interfaces.

The first aspect, chemical and structural specificities (primary to quaternary structures) of the two segments of an interface is poorly understood. The relation between the chemical/structural specificies of interfaces and the capacity of the two segments to recognize one another is also not clear.

Regarding this aspect, extensive work has been performed on the study of the sequence and/or structure of interfaces, particularly on dimeric interfaces [4], [5], [6], [7]; for a review see [8], [9], [10], [11]. The identification of protein interfaces has been based on several criteria such as solvent accessibility (ASA), distances cut-off, conservation residues [12], [13], [14]. When compared to interfaces found in crystallization induced dimers, some specificities were identified for true dimeric interfaces, such as larger interfaces, more hydrophobic residues and more conserved residues. Several databases dedicated to protein interactions or protein interfaces are available such as SCOPPI or PIBASE [15], [16], [17], [18]. The characteristic features of true dimeric interfaces fulfil the shape and the chemical complementarities required to form interfaces. More recently, programs combining both 3D structure analysis and multiple sequence alignments were able to identify so-called “hot-spot” residues or motifs as the crucial residues for the interface formation. These residues or motifs are selected by looking at conserved residues in interfaces of functionally related dimers, homologous or nonhomologous [15], [19]. However, interfaces seem to be only marginally residue specific and when that is the case it seems to reflect more a functional conservation of the residues rather than a true binding conservation [20]. Thus, the analyses of the sequences and of the 3D structures of protein interfaces reveal little on the chemical features that provide an interface with its capacity to associate.

The role of some of the amino acids of the interface in the formation of interfaces is known [21]. Thus, there is no doubt that the amino acids composition of the interfaces plays a role in the assembly process.

The geometrical treatment on α-helical coiled-coil protein oligomers and the knobs-into-holes model brought some light on how the “assembly capacity” of a protein is related to the sequence of the protein [22], [23]. The hallmark of coiled-coil sequences is the heptad repeat which is a contiguous run of a 7-residue consensus pattern of hydrophobic (H) and polar residues (P), HPPHPPP [24]. By convention, the residues of the heptad are labelled “abcdefg”. Hydrophobic residues tend to occupy the “a” and “d” sites so that in a repeated sequence they are alternately spaced three and four positions apart. Thus, when configured into a α-helix, which has 3.6 residues per turn, the “a” and “d” residues are brought together to set up a hydrophobic seam.

This illustrates that only few key amino acids of the interface (“a” and “d” in α-coiled) confer an oligomer its capacity to assemble. The chemical specificity of these amino acids (type of amino acids) is related to the geometry of the interface. This is why the oligomeric specificity of a protein cannot be distinguished by a trivial analysis of the whole sequence of the protein or the sequence of the interface. There are only few other examples of interfaces for which the relationship between the geometry and the sequence of the amino acids of the interfaces has been proposed, namely, the β-fiber (e.g. silk and spider web), the triple helix collagen and the β-spiral, which was more recently identified [25].

Concerning the mechanism of assembly, the mechanisms proposed to date, involve either the association of folded or almost fully folded monomers (induced-fit, lock and key and conformational selection) or the association of unstructured monomers (fly-casting) [26], [27], [28], [29]. But what brings a protein oligomer onto a particular assembly pathway remains unknown. Here, it is interesting to point out that proteins sharing the same assembly mechanism must share the elements (or at least some of the elements) responsible for the mechanism. Proteins with no obvious sequence homology and with different folds have been found to nevertheless follow the same assembly mechanisms [30], [31] (manuscript in preparation). On the other hand, some proteins with high sequence identity, similar structure and function might follow different assembly mechanisms [30] (manuscript in preparation). This indicates that only few amino acids among all that compose a protein are truly responsible for the mechanism and that there is no obvious strategy to search for them, like sequence or structural homology. Therefore, their identification is going to be extremely difficult and time-consuming, particularly if based only experimental approaches.

The third and last aspect important for understanding protein assembly is also not yet established. Experimental and molecular mechanic approaches, in particular on amyloid and on α-helical peptides, have revealed some of the necessary properties of a protein interface. Through the use of protein designs and binary libraries, it appears that a plethora of sequences provides a peptide its capacity to assemble. It is the sequential order of the chemical properties of the residues that generates a particular geometry and consequently provides the “association” capacity [32], [33], [34], [35], [36]. The formation of hydrogen bonding networks seems to be crucial whereas hydrophobic residues appear as the driving force of the assembly reaction [37], [38], [39]. Electrostatic interactions are identified as necessary for the maintenance of the association and seem to favor oligomerization against aggregation [40], [41]. Molecular mechanics also helps in identifying the role of amino acids in the assembly mechanisms [42].

Thus, it is clear that the elements (e.g. amino acids) that regulate the formation of interfaces are not to be considered as individual but as a network of interactions providing altogether an assembly of properties (hydrogen bonding, electrostatic and hydrophobic interactions) needed for association.

In summary, few amino acids are involved in the specificity of a protein interface and in the determination of the mechanism which leads to the formation of the protein interface. The chemical features of these amino acids must be consistent with the geometry of the interfaces. Finally these amino acids work together as a network. To assess these few amino acids and identify how they provide the interface its capacity to associate, we have developed a simple method which proposes a subset of the amino acids that are in a protein interface and which generates an interaction network of that interface.

We have developed a series of programs under the common name of Gemini, which isolates the amino acids involved in the protein interface of an oligomer from the rest, using the cartesian coordinates provided by the PDB database. Additionally, it can deal with a particular subset of “interacting” amino acids rather than identifying all the amino acids of the interface that can chemically interact. This procedure takes into account the observation that among the amino acids of an interface only few are truly crucial for its specificity and for its formation. Consequently, Gemini intentionally reduces the number of candidates “retained” as crucial for the interface. The selection is mainly geometrical (based on distances) with little selection on chemical properties of the amino acids or of the atoms. As a result, the method is extremely fast.

Despite the absence of a proper chemical screening, the results are remarkably accurate when compared to other programs. The speed makes the method suitable for comparison studies. The method generates a graphical representation of the 3D interface which is a comprehensible representation of the two segments of the interface and their possible interactions. This makes the method easily usable to design experiments (e.g. choice of amino acid to be mutated). Moreover, the graph constitutes an interaction network.

In summary, the aim of Gemini is to propose a framework of amino acid interactions involved in an interface so their role in providing the interface its specificity and in regulating the mechanism of assembly can be addressed, for example by comparing protein interfaces of similar geometry.

## Methods

A series of programs and database utilities have been created under the common name of Gemini to investigate properties of the interfaces of oligomer: GeminiDistances, GeminiRegions, GeminiGraph and GeminiData are of relevance for this paper.

### GeminiDistances

**Figure 1:**
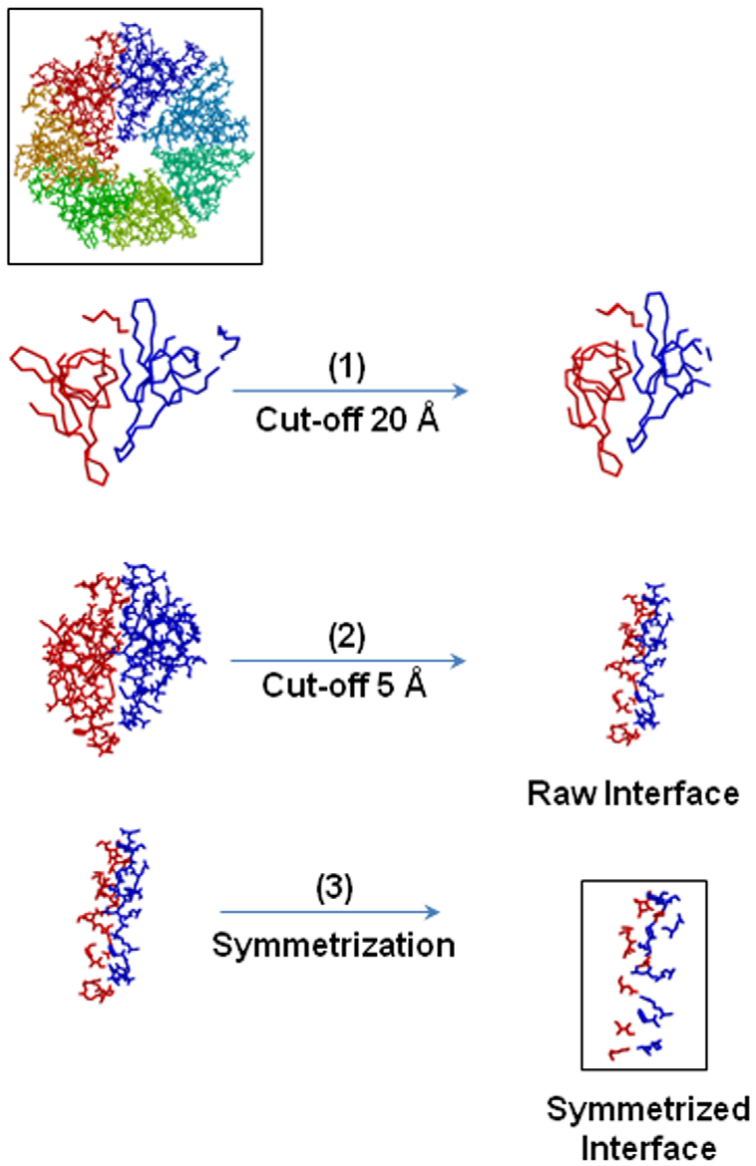
GeminiDistances selection. The heptameric cpn10 from *Mycobacter tuberculosis* (PDB code: 1HX5) is chosen to show the different steps performed by GeminiDistances to identify a subset of atoms involved in a protein interface [43]. The starting point is the complete atomic structure, shown in sticks, at the top. Each chain is indicated in a different colour. All the distances are calculated between pairs of atoms, one from chains A (blue) and one from chain G (red). The first step (1) selects the residues whose Cα are less than 20 Å apart. The second step (2) selects from all the atoms of step 1 those within 5 Å from one another. The third (3) and last step is a symmetrization identifying a unique distance which corresponds to the closest pair of atoms. The amino acids are indicated in backbone or in sticks when the Cα or all the atoms, are considered in the distance calculation, respectively. The output of step one and the input of step two coincide but the former is in backbone, the latter in sticks. The initial molecule and the final selected interface are highlighted in a black box.

This program has the main goal to recognize the interface between two adjacent chains M and M+1 in an oligomeric protein from its 3D structure as provided by the PDB file (http://www.rcsb.org/pdb/home/home.do). The objective is to find all pairs of atoms (one atom per chain) located at distances small enough for intermolecular interactions, and to reduce this set of interaction pairs to a minimum: the smallest set that still describes the protein interface. The idea is to generate a framework of the interface, made of a network of minimal interactions. A sketch of the procedure is illustrated in Fig. 1.

A first screening is done on the backbone Cα of the adjacent chains M and M+1: all pairs of amino acids (one per chain), whose Cα are separated by a distance lower than a given cut-off, fixed to “cut1 = 20 Å”, are retained for the next step, the others are discarded. This has the unique goal to speed up the calculation and is legitimated by the observation that the maximal amino acid theoretical length is about 8 Å. With much smaller distance cuts-off (e.g. 10 Å), some of the amino acids of the interfaces were missed.

In the second screening all the atoms of the amino acids previously retained are examined and the pairs at distance lower than “cut2 = 5 Å” are kept to form the so-called *raw interface*. This 5 Å distance covers the range of distances that corresponds to weak chemical bonds involved in interfaces: Van der Waals, electrostatics, hydrogen bonds [11]. Note that these cut-offs can be freely modified. The presence of the second cut-off, cut2, makes the raw interface de-facto independent of the first one: values of “cut1” of 17, 20, 25 Å and higher give identical results.

The raw interface is a long list of pairs of atoms that may form chemical bonds. For example, the interface of the heptamer co-chaperone 10, produced by *Mycobacter tuberculosis* (PDB code: 1HX5), has 328 pairs of atoms selected in the raw interface. These atoms correspond to 20 and 21 amino acids on the two adjacent chains, respectively. Because the aim of GeminiDistances is to propose a framework with a minimum of chemical interactions, it is necessary to add another constraint to the distance cut off of 5 Å to deselect a maximum number of pairs. The “deselection” is performed by a *symmetrization procedure* which only retains a single interaction per atom, the one involving the closest partner, even for atoms having more than one partner on the adjacent chain. Precisely, for each atom of M, in the raw interface, only the closest atom on M+1 is retained, yielding a set of pairs L1. Similarly, for each atom of M+1, in the raw interface, only the closest one on M is retained to form a second set of pairs L2. The pairs common to both lists (L1 intersection L2) form the interface used for the investigations of this paper, and is also called *symmetrized interface*. In other words, a pair of atoms (i, j) is in the interface if both “i” is the closest to “j” and “j” is the closest to “i”.

The symmetrization makes the symmetrized interface almost cut-off independent. Indeed, we have explored values in the range “cut2”  = 4.5 to 6 Å. For “cut2”  = 4.5 Å, some interactions are lost and the raw interface forms a subset of the raw interface obtained with “cut2” = 5 Å. Vice versa for “cut2”  = 6 Å the raw interface is bigger. After symmetrization, we observe remarkably small variations: in average, they do not exceed 10% of the interface in the indicated range for cut2. Variations are even smaller if only amino acids and not atoms are considered.

It is important to keep in mind that the symmetrization discards many atoms at distances for which a chemical interaction is plausible. Therefore, the output generated by GeminiDistances may miss atoms and amino acids (false negative). It may also select atoms which are not chemically the most plausible (false positive). But the selection of the most chemically plausible interactions is a more difficult task than the geometrical selection performed by GeminiDistances. A more chemical selection would be necessarily slower and might not necessarily be more accurate. Such a method may be better for a case-to-case study, but the symmetrization is more appropriate for a rapid comparison of the interfaces of many oligomers. GeminiDistances contains an option so it can work with or without the symmetrization. Thus, the entire set of amino acids involved in the interfaces (cut2) or only a subset of these amino acids (cut2 + symmetrization) can be considered, according to the user's need.

**Figure 2:**
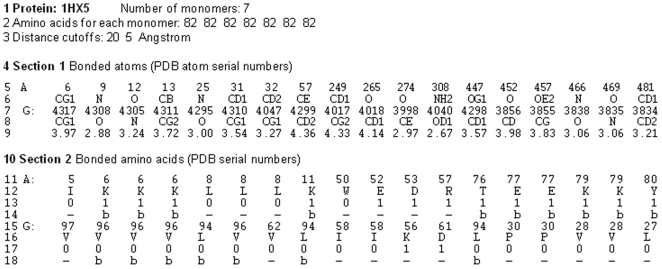
Output generated by GeminiDistances. Here we give the interface of the heptameric co-chaperonin 10 from *M. tuberculosis* (PDB code: 1HX5) [43]. The first line gives the PDB code and the stoichiometry of the protein oligomer. The second line indicates the total number of amino acids seen for each individual chain on the x-ray structure. Section 1 (lines 4-9) contains the pairs of interacting atoms in the interface. In section 2 (lines 10 -18), the corresponding amino acids are indicated. The pairs of atoms (or of amino acids) are shown on the same column. The atoms of the two adjacent chains A and G are given on lines 5-6 and 7-8, respectively. For each chain, the first line indicates the PDB progressive number of the atoms (lines 5 and 7 for chain A and G, respectively) and the second line indicates the type of the atom (carbon, oxygen…) and its position within the amino acid (lines 6 and 8 for chain A and G, respectively). The line 9 indicates the atomic distance (in angstroms) between each pair of atoms. The same format is used in section 2 and the information on the amino acids for the two adjacent chains A and G are given on lines 11-14 and 15-18, respectively. The progressive number of the amino acid along the sequence, is given on the line 11 followed on the next line by the type of amino acid. Lines 13 and 17 indicate the hydrophobicity of the amino acid (1 and 0 for hydrophilic and hydrophobic, respectively); lines 14 and 18 give the secondary structure of the amino acid, according to the x-ray structure.

**Figure 3:**
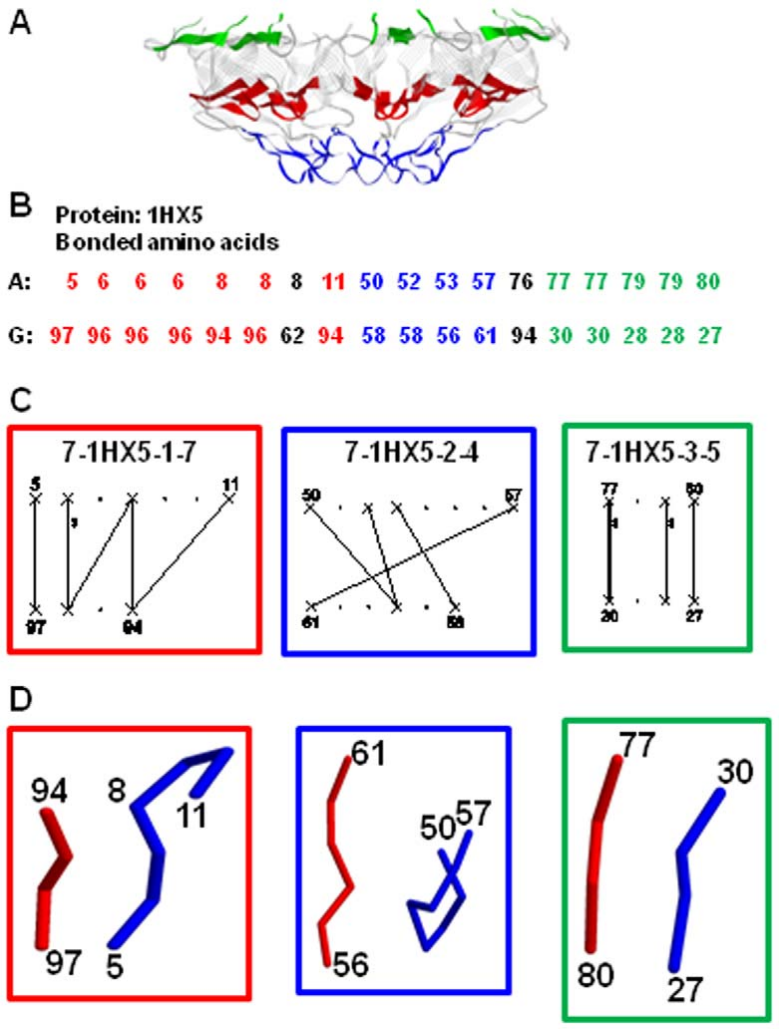
Interaction network. **A.** x-ray structure of the heptameric co-chaperonin 10 from *M. tuberculosis* (PDB code: 1HX5) [43]. GeminiRegion detects three different regions of the interface of 1HX5, indicated in different colors. **B. Section 2 of the GeminiDistances output of the 1HX5 interface.** Only the amino acids of the two adjacent chains are indicated. The color code is the same as in 3A. **C. Interaction networks of 1HX5.** Based on GeminiDistances and GeminiRegion (see methodology), GeminiGraph visualizes each region of the interface as a graph, referred to as an interaction network. 1HX5 has three regions of interface and therefore three different interaction networks, indicated in boxes matching the colors of 3A. The interaction network labels give: first, the stoichiometry, second the PDB code, third the number of the region and last the number of the “framework chemical bonds” according to GeminiDistances. The amino acids that compose the segments of the interface are indicated on two parallel lines. For each segment, the amino acids involved in a chemical bond are symbolized with an “X” whereas the others are symbolized with a dot “.”. The chemical bond that connects two “X” is symbolized by a continuous line, called a link. The number of chemical bonds connecting two amino acids is symbolized by a number on the right of the link and by the thickness of the link. The sequence number of the amino acid is indicated above (or below) the corresponding “X”. **D. The 3D structure of the segments that compose the interface regions, as seen on the x-ray structure.** Each 3D structure is framed following the same color code of 3A. For each region, the segments of chain A and G are indicated in red and blue backbones, respectively. Some of the amino acid sequence numbers are indicated. The images of the x-ray structures were generated using Rasmol.

From the 328 pairs of atoms selected for the raw interface of 1HX5, only 18 pairs remain after symmetrization (Figs. 2 and 3). In a more coarse grained interpretation, the atoms of the symmetrized interface are replaced by the amino acids they belong to. This *amino acids interface* is used by the next program GeminiRegions.

In the paper, the search for the interface has been done on a single pair of adjacent chains. A full search on all chains is also possible and necessary when non-circular oligomers are considered.

GeminiDistances is written in C and runs in less than 0.3 s for an average size protein, on a normal desktop computer.

### GeminiRegion

This program separates the amino acids interface, given by GeminiDistances, into regions, termed as elementary *interaction networks* between the amino acids of two adjacent chains. An example is given for the interface of the heptamer co-chaperone 10 from *M. tuberculosis* in figure 3. Here, the interface is composed of three physically separated regions illustrated by three different colors, regions 1 (red), 2 (blue) and 3 (green), in Figure 3A.

Two pairs of amino acids in the amino acids interface, (A1,A2) and (B1,B2), are considered to belong to different regions if their respective amino acids on one segment are more than 5 amino acids apart from each other along the sequence: if one of |A1−B1|>5 or |A2−B2|>5 the pairs are put in different regions (1 and 2 representing two adjacent chains). In addition, regions with just one pair are ignored and the corresponding amino acids are ignored.

This algorithm expresses the concept that amino acids in a region must be “close” along the sequence, in addition to be “close” in space as considered in the construction of the interface itself (example in Fig. 3B). By construction, a region, or an interaction network, contains the interactions expressed by the pairs of the amino acids that form the interface; this corresponds to the notion of *graph*. In mathematics, a graph is a set of vertices (here the amino acids) connected by a set of links (here the weak chemical bonds).

This C++ program runs in the infinitesimal time of 2 ms per protein.

### GeminiGraph

This program creates a graphical representation of the interface regions and their amino acids interactions in the style of graph theory. Here the vertices are the amino acids; those involved in a weak chemical bond are symbolised by a cross “X” whereas those not involved in weak chemical bonds are symbolized by a dot “.”. Unlike GeminiRegion and GeminiDistances, GeminiGraph needs to consider all the amino acids of the segments and not only those selected as chemically interacting. Technically, this is called a bi-colored graph (“X” is the first color and “.” is the second color).

Links (continuous straight lines, vertical or oblique) are inserted when a weak chemical bond is present between amino acids belonging to different segments of an interface region namely, between amino acids represented by a “X”. By convention, the amino acids of a segment are equally spaced on an horizontal straight line. The two segments which form a region are represented by two parallel horizontal lines systematically positioned at a fixed distance, independently of the real atomic distance that separated the pairs of atoms (Fig. 3C). The resulting graphical representation is topological, namely it keeps track of connections but not of physical distances.

The numbers of the first and last amino acids of each of the segments are indicated above the appropriate line. The number of chemical bonds per amino acid is indicated by a number on the right side of the link. For example, the amino acids (6, 96) (Fig. 3C, red interface) are connected to one another by three chemical bonds, identified by the number 3 on the right side of the link. The thickness of the link also increases with the number of chemical bonds per amino acid. Each interaction network is labeled with the stoichiometry of the protein, its PDB code, the progressive number of the region and finally the total number of chemical bonds involved in that region.

### GeminiData

This database faciliates the access to the relevant protein data, including the results of GeminiDistances, GeminiRegions and GeminiGraph. The full amino acids sequences and other useful information are included, for examples: chemical properties of the amino acids and 2D structure when available. The database is still under constrution and is accessible by the language MySQL.

## Results

### Gemini

The 3D structures of all the oligomeric proteins whose atomic structure is available are considered in our study. The goal of our work is to establish a method that (i) extracts only the atoms involved in the interface, using the 3D x-ray structure, (ii) proposes a subset of interacting atoms and (iii) generates a schematic representation of the resulting interaction network.

The output generated by GeminiDistances is standardized to the format shown in Figure 2; only specific information is extracted from the PBD file of the atomic structures of protein oligomers to accompany the interface data.

The application of Gemini to the heptamer co-chaperone 10 (PDB code: 1HX5), produced by *Mycobacter tuberculosis*, is indicated in Figure 2 [43]. The first line gives the PDB code and the stoichiometry of the protein oligomer (number of chains forming the oligomer). The second line indicates the total number of amino acids seen for each individual chain on the x-ray structure. Section 1 (lines 4–9) contains the pairs of atoms selected by GeminiDistances as interacting in the interface. In section 2 (lines 10–18), the corresponding amino acids are indicated. The information on the atoms of the two adjacent chains A and G are given on lines 5–6 and 7–8, respectively. For each chain, the first line indicates the PDB progressive number of the atoms (lines 5 and 7 for chain A and G, respectively) and the second line indicates the type of the atom (carbon, oxygen…) and its position within the amino acid (lines 6 and 8 for chain A and G, respectively). The line 9 indicates the atomic distance (in angstroms) between each pair of atoms. The pairs of atoms (or of amino acids) are shown on the same column. The same format is used in section 2 where the information on the amino acids for the two adjacent chains A and G is given on lines 11–14 and 15–18, respectively. The progressive number of the amino acid along the sequence is given on the line 11 followed by the next line by the type of amino acid. Lines 13 and 17 indicate the hydrophobicity of the amino acid (1 and 0 for hydrophilic and hydrophobic, respectively); lines 14 and 18 give the secondary structure of the amino acid, according to the x-ray structure.

The PDB contains about 4000 different atomic structures of oligomers, considering the stoichiometries from trimer to dodecamer (Table 1). Oligomers with factorized stoichiometry (e.g. 6 = 2×3 or 9 = 3×3) appear more numerous than nearby prime-number stoichiometries (e.g 3, 5, 7) because they often are the result of an arrangement of lower stoichiometries. For example, an hexamer can be formed by two trimers or three dimers or by a circular hexamer. Consequently the total number of structures of factorized stoichiometry is higher. The circular symmetric cases cannot result from the combination of lower stoichiometries and a decreasing behaviour appears (Table 1).

**Table 1:** Total number of protein oligomers as determined from the pdb.

**Stoichiometry**	3	4	5	6	7	8	9	10	11	12	Total
**Total Proteins**	780	1764	102	491	26	307	20	56	2	116	3664
**Circular Proteins**	339	39	54	43	22	5	2	1	1	1	507

Given the large number of data, a database (GeminiData) was created to store protein and interface data, in particular the GeminiDistances and GeminiRegions outputs. So far, 2843 oligomers have been stored.

Using GeminiData, it has been determined that in average, the oligomers of our dataset contain 250 amino acids (717 075 amino acid for 2843 protein oligomers) per chain. This is in good agreement with other estimations that found an average length of about 300 amino acids [1], [44].

The percentage of amino acids involved in interfaces for each oligomer is calculated as the ratio of the number of amino acid selected by GeminiDistances to the total number of amino acids of the protein. An average, per stoichiometry, is made on all the protein oligomers, and not only on circular ones (Table 2). In average about 20% of the protein oligomer residues participate in interfaces for all the stoichiometries considered, except for the stoichiometry 11 for which this figure deviates significantly. This probably results from the average made on a smaller number of cases. It is important to note that for each stoichiometry the standard deviation is extremely high. Thus, there is an important variability in the number of amino acids that participates in protein interfaces.

**Table 2:** Percentage of amino acids involved in interfaces.

**Stoichiometry**	3	4	5	6	7	8	9	10	11	12
**Percentage of interfacial residues**	18±16	18±15	22±17	19±8	25±15	19±17	13±11	23±11	46±6	21±17

The stoichiometry 11 is not considered further in the study as the number of available atomic structures is too small.

**Figure 4:**
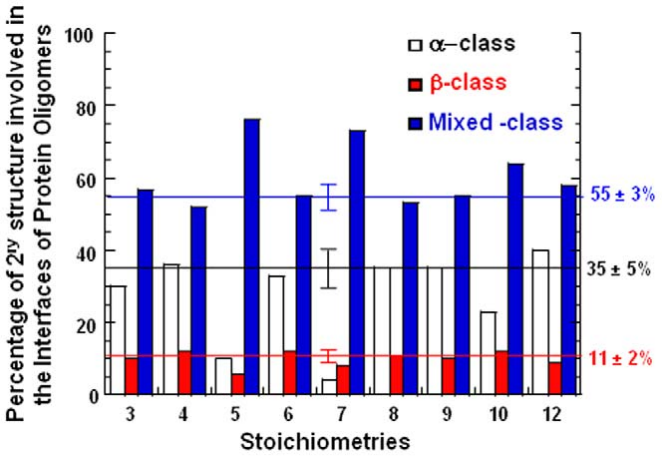
Secondary structure distribution per stoichiometry. The percentages of the secondary structures observed in protein interfaces are plotted against the stoichiometry. Three classes of secondary structures are considered, pure α (white bar), pure β (red bar) and mixed (blue). For each class, the average across the different stoichiometries is indicated on the right of the plot and illustrated by a straight line.

Based on the GeminiDistances output, the 2D structures of the interfaces of all oligomers are analyzed and divided in three classes (Fig. 4). The α-class is composed of oligomers whose interface uniquely involves α-helices on both the segments of the interface. Similarly, the β-class is composed of oligomers whose interface uniquely involves β-structures on both the segments of the interface. All the rest are grouped into the mixed-class. The protein distribution in the various classes is calculated as the ratio of the number of protein oligomers of one class (e.g. α-class) to the total number of protein oligomer (α-class +β-class + mixed-class). In Figure 4, the percentage of each class is plotted against the stoichiometry. Manifestly, the distribution of the secondary classes is similar throughout most of the stoichiometries (3, 4, 6, 8, 9, and 12) with about 33% of the interfaces composed of α-helices, about 10% of β-structures and about 56% of mixed structures. However, there is an apparent difference for the stoichiometries 5 and 7 for which almost 80% of the interfaces are made of mixed structures. It is also true, although to a lesser extent, for the stoichiometry 10.

Based on the GeminiData, the percentage of polar residues present in interfaces of all oligomers is calculated as the ratio of polar amino acids present in an interface to the total number of amino acid of that interface (Table 3). The figures are similar throughout the different stoichiometries with an average value of about 57±15%.This is more than the average value of 35% estimated for homodimers but it is in relatively good agreement with the average value of 44% estimated for other interface categories [14], [45].

**Table 3:** Ta Percentage of polar residues in interfaces.

**Stoichiometry**	3	4	5	6	7	8	9	10	12
**Percentage of polar residues**	58±15	58±17	56±13	58±16	55±12	57±17	63±15	52±13	57±16

The percentage of interfacial residues, of polar residues present in interfaces and the distribution of the secondary structures in interfaces are similar when only the circular oligomers are considered (not shown).

### GeminiRegion and GeminiGraph

GeminiDistances is capable of extracting well-defined information relevant to describe interfaces. Interfaces are often made of several physically separated regions, which need to be considered individually. For this purpose, GeminiRegions was developed. It inputs GeminiDistances interfaces and recognizes each individual region (see the Methodology for the details of the procedure). For example, the interface of the heptamer 1HX5 is made of three physically separated regions of amino acids (Fig. 3A), regions 1 (red), 2 (blue) and 3 (green), which have been easily identified by GeminiRegions (Fig. 3B).

For the analysis of each region of an interface, GeminiGraph was developed. Using the GeminiRegion output, it creates a graphical representation of each region of the interface (interaction network), inspired from the graph theory. The three interaction networks corresponding to the three regions of 1HX5 are indicated in Figure 3C. The detailed description of GeminiGraph and of the interaction networks are given in the Methodology and in the legend of figure 3.

In brief, the amino acids of each segment are indicated on a line. Those selected as interacting (i.e. involved in a weak chemical bond) are represented by “X” and the others by a dot “.” (e.g. residues 5 and 7 of the red interface in Fig. 3C are X and, respectively). Each interaction network is labelled with a title which indicates the stoichiometry of the protein followed by its PDB code, the number of the region and finally the total number of “bonds” in the region, as identified by GeminiDistances. For instance, 1HX5 has three regions, with 7, 4 and 5 bonds, respectively.

### Interaction networks

To validate the methods of GeminiDistances and GeminiRegions, it is necessary to determine if the interaction networks reflect the structures of the interfaces obtained by examination of the PDB atomic coordinates and, if yes, how that information is expressed on the graphs. This will be called geometrical validation. It is also important to evaluate the chemical accuracy of the selection performed by GeminiDistances, particularly to determine the validity of the symmetrization procedure. We will call it chemical validation.

### Geometrical validation-1

The 3D arrangement of the three regions of interface of 1HX5 is shown in Fig. 3D below their relative interaction network (Fig. 3C). According to the x-ray data, the structures of both the red box and the green box regions are rather similar as they are composed of two aligned β-strands, one per segment. Now, according to GeminiDistances, the red box and the green box regions also have rather similar interaction networks (Fig. 3C): they share a common domain composed of two *blocks* of sequence “XX. X”, one per segment, interacting with each other (vertical links in Fig. 3C). In the red box region, there is an additional loop at the C-terminal extremity of one of the β-strands (Fig. 3D) that is compatible with the additional domain represented in the graph with the two blocks of sequences “X. X” and “X. X” interacting with each other (oblique links in Fig. 3C). These additional blocks are absent in the green region.

The 3D structure of the blue box region is significantly different from those in the red and green boxes as are different the corresponding interaction networks (Fig. 3D and 3C, respectively). It is interesting to note that the block “XX. X” is also present in the blue region but only on one of the segments of the interface, so the two-block interaction is now different from the one seen in the red box and the green box regions. This suggests that features of the 3D geometry of the interface are found in interactions between blocks and not in individual blocks.

### Geometrical validation-2: the 3D geometry of interfaces

As mentioned in the methodology, in the graphs generated by GeminiGraph the distance between two interacting segments of interface is fixed and identical for all interfaces, independently of the real atomic distances that exist between the interacting atoms. So the length of the links has no special meaning.

Yet, in reality, the bonded atoms that are retained by GeminiDistances are separated by a distance not longer than 5 Å and two α-carbons of contiguous amino acids on a segment are separated by no more than 4.5 Å (evaluated on the average radius of covalent bonds in proteins). This implies that the “topological” information of the graph yields geometrical constraints on the corresponding 3D structure. For example, residues 8 and 11 are both interacting with residue 94 on the adjacent chain (Fig. 3C, left panel). For this to happen, either residues 8 and 11 are sufficiently close in space, which implies some curvature in the backbone, or the side chains of residues 11 and 94 are long enough to permit an interaction between their atoms. If the lengths of the side chains are too small, the backbone is necessarily curved. However, long enough side chains at that position do not allow us to discriminate *a priori* between the two options. Here, this is precisely the case as residues 11 and 94 are a lysine and a leucine respectively, both amino acids with long side chains, yet the backbone connecting residues 8 and 11 is curved (Fig. 3D). This shows that the deconvolution of the 3D information present in the topology of the graph is not trivial.

### Geometrical validation-3: the 2D structure of interface

**Figure 5:**
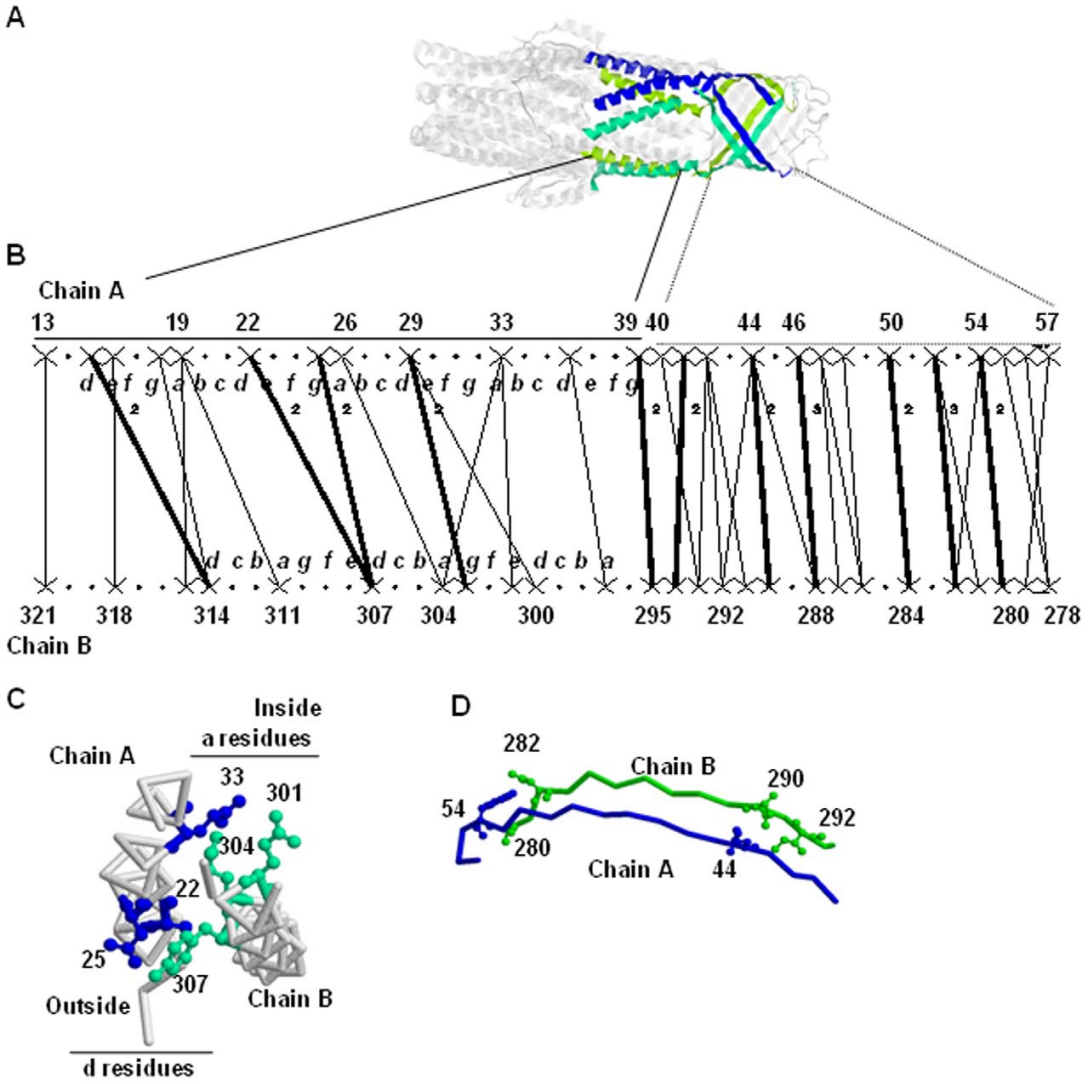
Interfaces of the trimeric membrane protein Tol C (PDB code: 1EK9). A. x-ray structure of TolC [47]. The three monomers are indicated in three different colors. This protein contains both α- and β- interfaces. The α-region is composed of chemical bonds between residues 13 to 39 of chain A and residues 295 to 321 of chain G. The β-region is composed of residues 40 to 57 of chain A and residues 278 to 294 of chain G. B. Interaction Networks of the α-region (left) and of the β-region (right). The “a” to “g” heptad repeat [48], which is conventionally used to describe α-coil interfaces, is reported on the segments. C. The 3D structure of the α-region. Some of the amino acids forming knobs-into-holes are indicated in balls and sticks. They are colored using blue and green for chains A and B, respectively. D. The 3D structure of the β-region. The amino acids involved in chemical bonds are indicated in balls and sticks. The images are generated using Rasmol. The symbols are described in Figure 3 and in methodology.

We then investigate whether the 2D geometry of the interface is also indicated in the interaction network. We look at the interface of the trimeric membrane protein TolC (PDB number: 1EK9) because it has both α-helical and β-sheet interfaces as can be seen on its x-ray structure (Fig. 5A) [46]. The region composed of the two segments from residues 13 to 39 of chain A and from residues 295 to 321 of chain G is a α-helical interface. The region composed of the two segments from residue 40 to 57 of chain A and from residue 278 to 294 of chain G is a β-sheet interface. The interaction networks of the α and β regions are shown in Fig. 5B, on the left and right panels, respectively. One can see that the α–and the β–regions have their own particular sequences of “X” and “.”. More precisely, the two interaction networks have very dissimilar ratio of interacting “X” amino acids by the total number of amino acids in the interface region. Indeed, there are 12 “X” amino acids out of 27 that make up each of the two segments of the α-helical region but there are 13 and 14 interacting “X” amino acids out of 18 and 17 that make up the chain A and B segments respectively, for the β-region. This means that the α-helical region contains 44% “X” interacting amino acids against 77% of the β–sheet region (actually 72 and 82% for chain A and B, respectively). This difference is due to their 2D geometry. To make a contact surface of “X” amino acids, approximately twice total amino acids are necessary with a β–geometry than for a α–geometry. Two amino acids come on the same face about every 3.6 amino acids for a α–helix but every alternate amino acid for a β-sheet structure. Hence, to get a good estimate of the 2D geometry of the interface from the interaction network, one can calculate the ratio of “X” to the total number of amino acids of one segment. Other α-coiled (5 cases) and 2β-strand interfaces (10 cases) have been analyzed (not shown), and in average, the α-coiled interface regions contain 37±14% amino acids “X” per segment whereas the β-sheet regions contain 66±25% interacting “X” amino acids per segment.

Thus, as observed for the interfaces of 1HX5, the interaction networks from GeminiDistances contain strong indications about the 2D structure of the interface.

Another noticeable difference between the α-helical and the β–sheet regions is in the number of amino acids that separate two amino acids of one segment interacting with the same amino acid on the other segment. This number is referred to as the sequence distance or *1D distance*. For example, on the α-helical interface, the residue 33 of chain A interacts with both the residues 301 and 304 of chain B (Fig. 5B) with a typical knobs-into-holes structure (Fig. 5C) [47]. The residue 33 is the knob and the hole is made of the residues 301 and 304 of the adjacent chain G (Fig. 5C). There are five other knob-into-holes cases easily identified on the interaction network (Table 4). In all the cases, these pairs of amino acids are 3, 4 or 7 amino acids apart, corresponding to one or two helix turns, given an average turn of 3.6. The recognition of these pairs of amino acids and their counterpart (on the opposite segment) in both the chains of the graph indicates that the interface region is made of two interacting α-helices.

**Table 4:** Pairs of amino acids and their common interacting counterpart.

Chain A	Chain B	Linear Distance	Chemical properties
P15, R18	E314	3	f, Ch, Ch
A22, D25	Y307	3	f, Ch, P
A26, E33	Q304	7	f, Ch, P
K19	G311, G315	4	Ch, f, f
E29	S300, K303	3	Ch, P,Ch
E33	Q301, Q304	4	Ch, P, P

As already reported by Calladine and co-workers, the residues that compose the knobs-into-holes framework of the TolC α-interface have some chemical specificity. They are either small (A, S, G, C) or polar with long side-chains (D, E, K, R, H, Q, N) [47].

A similar analysis was performed for the β-sheet region and 12 groups of two amino acids and the same counterparts are found in both the chains (Table 5). In all the cases, these groups of two are 1 or 2 amino acid apart, which indicates that they are on a β-strand. The backbone of a β-strand is arranged in a zigzag fashion (pleated) and consequently, the side chains of two neighbouring residues project in opposite directions. So due to this particular geometry, it is the side chain of alternate residues that forms the interaction between the β-strands. Sometimes, the pleated geometry is not perfect and the backbone of two contiguous amino acids of the same strand can be in the same plan. So, the analysis of the interaction network of the β-region enables us to conclude that the two segments are made of two rather aligned β-strands.

**Table 5:** Pairs of amino acids and their common interacting counterpart.

Chain A	Chain B	Linear Distance	Chemical properties
L42	Y293, I292	1	f, P, f
L42	I292, P291	1	f, f, f
L44	I292, L290	2	f,f,f
L44	L290, F288	2	f, f, f
D47	L286, S287	1	Ch, f, P
N52	Q281, N282	1	P, P, P
Y54	G280, N282	2	P, f, P
P40, L42	Y293	2	f, f, P
L42, L44	I292	2	f, f, f
L44, A46	F288	2	f, f, f
D47, Y48	L286	1	Ch, f, P
D56, R55	N278	1	Ch, Ch, P

Thus, an alternative method to identify the 2D structure of a protein through the interaction network is to count the sequence distance (number of amino acids apart) between amino acids of the same chain sharing the same partner. The distances are of 3–4 amino acids for α-helical structures or of 1–2 amino acids for β-sheet structures.

Lastly, it is necessary to evaluate the chemical validity of the amino acids “X” selected as interacting by GeminiDistances. This is particularly important as the symmetrization provides a unique pair of interacting atoms, and hence of interacting amino acids, from all the chemically possible pairs. The pairs selected are the atoms at the shortest distance. Therefore the entire procedure is purely geometric, namely distance based, and there is no selection based on the chemical properties of either the atoms or of the amino acids, apart from the cut2 of 5A. On given examples, the selection performed by GeminiDistances will be compared with that obtained with others methods to evaluate the chemical accuracy of the symmetrization. The false negatives and the false positives, which are the amino acids detected as involved in a chemical interaction by other methods but not by GeminiDistances and the way around, respectively, are calculated as an estimate of the validity of the symmetrization.

### Chemical validation-1

Using PDB viewer, the amino acids involved in hydrogen bonds have been computed for the interfaces of a set of 40 proteins (not shown). We have then checked whether those amino acids were selected as “X” by GeminiDistances. About 87% of the PDB computed hydrogen bond amino acids are correctly selected as “X” by GeminiDistances. It actually identifies more amino acids than those computed by PDB viewer, as expected since there are other possible weak bonds.

### Chemical validation-2

Using again the interface of the trimeric membrane protein TolC, we have compared the amino acids detected as interacting by GeminiDistances and by two other programs, namely PPIDB and SCOWLP [48], [49]. The PPIDB selection is based on atomic distances with a cut-off at 5 Å and the SCOWLP detection is based on atom types and distance criteria. The segments composing the TolC interface are identified likewise by the three programs and are made of residues ranging from 13 to 57 and 278 to 321 for the chain A and B, respectively. One additional residue, the 12, is detected only by PPIDB. Out of the 89 residues composing the interface, 51, 55 and 66 respectively are detected as interacting “X” by GeminiDistances, SCOWLP and PPIDB, respectively. Among the 51 detected by GeminiDistances, 47 and 41 are commonly identified by SCOWLP and by PPIDB, respectively. Therefore, there are 4 and 10 false positive residues compared to SCOWLP (51 minus 47) and to PPIDB (51 minus 41), respectively. Similarly, GeminiDistances misses 8 and 25 false negative residues compared to SCOWLP (55 minus 47) and to PPIDB (66 minus 41), respectively. Out of the 55 residues detected as interacting by SCOWLP, 45 residues are also identified by PPIDB. Hence, SCOWLP identifies 10 (55 minus 45) false positive residues and 21 (66 minus 45) false negative residues compared to PPIDB. Clearly, the selections of GeminiDistances and of SCOWLP are closer to each other than to the selection of PPIDB.

### Chemical validation-3

As mentioned in the introduction, α-coiled interfaces have been well-studied and described as heptad repeats labelled from “a” to “g” (see the review in [23]). The residues that form the interaction core between the coiled α-helices are the “a” and the “d”. Accordingly, the amino acids at these particular positions have some chemical specificity (type of amino acids) [47]. The trimeric membrane protein TolC forms a 12-helix hollow cylinder, referred to as a α-barrel [46]. Calladine and co-workers have described the TolC α-coiled interface as constituted by the “a” residues, towards the inside of the channel, and by the “d” residues, towards the outside of the channel (Fig. 5C, left panel). Accordingly, the inside core (residues “a”) is formed through interactions between the following amino acids of chains B and A, respectively: (297, 33), (304, 33), (304, 26) and (311, 19). Similarly, the outside core (residue “d”) is made of interactions between the amino acids of chains B and A, respectively: (300, 29), (307, 22) and (314, 15).

Using the heptad repeat nomenclature of α-coiled (Fig. 5B, left panel), we could check that GeminiDistances detects successfully the 13 “a” and “d” residues as interacting “X” amino acid (Fig. 5B). Moreover, GeminiDistances is also able to detect 6 out of the 7 chemical bonds between these residues, only the chemical bond between residues 297 and 33 of chain B and A, respectively, is not detected, the residue 297 is detected interacting with residue 36 instead (Fig. 5B). This gives a successful detection rate of 87%.

The detection of “a” and “d” residues as “X” interacting amino acids has been analyzed for three other α-coiled interfaces (not shown) and in average there is below 5% of false-negative.

Beside the “a” and “d”, other residues are detected as interacting “X” by Geminidistances, which is consistent with the fact that other residues are known to be involved in interactions in α-coiled interfaces [50], [51], [52]. However, because these other interactions are not systematic, it is not possible to estimate the amount of false positives among the additional “X” detected by GeminiDistances.

### Chemical validation-4

**Figure 6:**
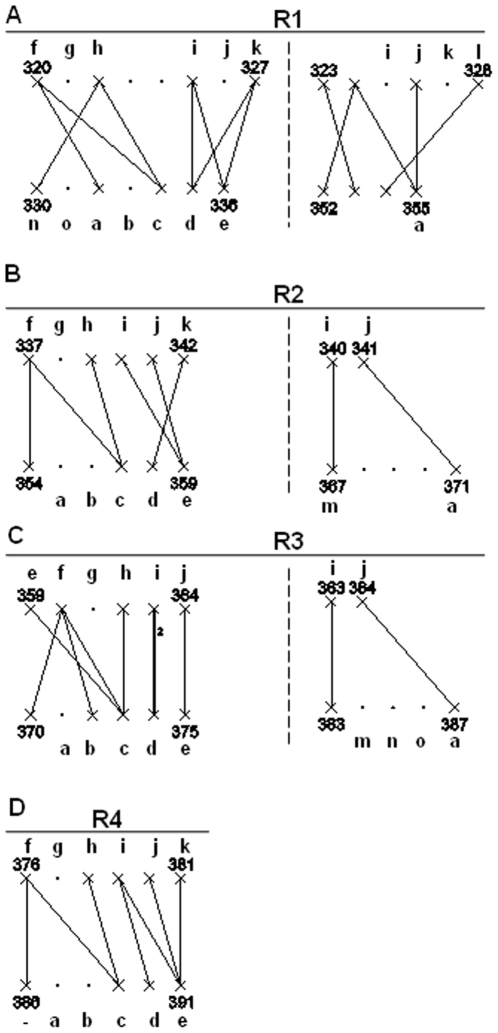
Interaction networks of the four pseudo-repeats of the shaft domain of the adenovirus type 2 (PDB code 1QIU) [**25**]. A. First pseudo-repeat. B. Second repeat. C. Third repeat. D. Fourth repeat. According to van Raaij and co-author, each interface region involves chemical bonds between residues of the pseudo repeat Ri of one chain and residues of the pseudo-repeat R'i of the adjacent chain. These chemical bonds are observed on the interactions on the left of the figure. There is an additional chemical bond between residues of the pseudo repeat Ri of one chain and residues of the pseudo-repeat R'i+1 of the adjacent chain. This chemical bond is observed on the interaction networks on the right of the figure. Each pseudo-repeat is made of 15 residues named “a” to “o”, as described by van Raaij and co-authors. There are hydrophobic interactions between residues “c”, “k” and “e” and hydrogen bonds between residues “c” and “f”; “c” and “h”; “e” and “I”, “a” and “j”. The “a” and “j” chemical bond belong to the Ri, R'i+1 interaction networks. The images of the x-ray structure are generated using Rasmol. The symbols are described in Figure 3 and in methodology.

To test the GeminiDistances program further, we have generated the GeminiDistances output for the β-spiral trimeric interfaces of the shaft domain of the adenovirus type 2 (PDB code: 1QIU), for which the key amino acids involved in the interfaces have been well described [25]. The interface of the shaft domain is made of 22 pseudo-repeats of 15-residues (“a” to “o”) sharing consensus sequences. Only four of these 22 pseudo-repeats are seen on the x-ray structure of the shaft domain and they share very similar 3D structures. Each chain of the trimer is composed of the four pseudo-repeats {R1, R2, R3, R4}. The interface is made of chemical bonds between R1 and R1′ and between R1 and R2′ where the prime distinguishes different chains; in general, the interface is made of bonds between Ri and Ri' and between Ri and Ri+1′. According to van Raaij and co-authors, at positions “c”, “e”, and “k” there are hydrophobic residues forming the hydrophobic interaction core, whereas pairs (f, c), (h, c), (j, a), (i, e) are involved in an hydrogen bonding network. Residues “c” and “e” are involved in both the hydrophobic and the hydrogen bonding networks. All the chemical bonds are between Ri and Ri' apart from (j, a) which takes place between Ri and Ri+1′ (See Fig. 3 in [25] and Fig. 6).

For the first repeat R1 (Fig. 6A), 9 amino acids were detected as “X” by GeminiDistances out of the 9 identified by van Raaij and co-authors. The chemical bonds (f, c), (h, c), (i, e) and (k, e) are also detected properly on a single interaction network (Fig. 6A, left). The hydrophobic interaction (k, c) is a false negative, undetected. The chemical bond (j, a) is detected on a separated interaction network (Fig. 6A, right) consistently with van Raaij and co-authors description of the interfaces R1/R′1 and R1/R′2. GeminiRegion distinguishes the two interaction networks R1/R′1 and R1/R′2 because two consecutive chemical bonds must be less than 5 amino acid apart on both the chains simultaneously to be considered in the same network. There are also 3 false positive chemical bonds generated by GeminiDistances, involving the pair (322, 330) of type (h, n), the pair (320, 332) of type (f, a) and the pair (325, 335) of type (i, d).

A similar analysis has been performed for the three other repeats with similar results. For the second repeat R2 (Fig. 6B), 8 amino acids out of the 8 identified by van Raaij and co-authors, have been detected as interacting residues “X” by GeminiDistances. The chemical bonds (f, c), (h, c) and (i,e) are detected properly on the same interaction network (Fig. 6B, left) and the chemical bond (j, a) is detected on a separate interaction network (Fig. 6B, right). The hydrophobic interactions (k, c), (k, e) remain unidentified (false negatives). In addition, there are three false positive chemical bonds detected, namely (337, 354) of type (f, 354), (342, 358) of type (k, d) and (341, 371) of type (j, a). In the former case, the residue 354 does not belong to the “a-o” repeat of van Raaij and co-authors.

For the third repeat R3 (Fig. 6C), there are again 8 amino acids detected as interacting residues “X” by GeminiDistances out of the 8 identified by van Raaij and co-authors. The chemical bonds (e, c), (f, c) and (h, c) are detected properly on the same interaction network (Fig. 6C, left) and the chemical bond (j, a) is detected on a separated interaction network (Fig. 6C, right). There are four false positives detected, (360, 370) of type (f, 370), (360, 372) of type (f, b), (363, 374) of type (i, d). In the former, the residue 370 does not belong to the “a-o” repeat of van Raaij and co-authors.

For the fourth repeat R4 (Fig. 6D), there are again 8 amino acids detected as interacting residues “X” by GeminiDistances out of the 8 identified by van Raaij and co-authors. The chemical bonds (f, c), (h, c), (k, e) and (i, e) are also detected properly on the same interaction network. However, the hydrophobic interaction (k, c) is not identified. The shaft stops at residue 393, from which the Ad2 head domain starts, so there is no possible (j, a) bond for this last repeat. There are three false positive chemical bonds detected, (376, 386) of type (f, 386), (380, 391) of type (j, e), (379, 390) of type (i,d). In the first case, the residue 386 does not belong to the “a-o” repeat of van Raaij and co-authors.

On average, on the four repeats GeminiDistances is able to detect 85% of the amino acids involved in a chemical interaction (28 “X” detected onto 33 total) and it is also able to identify 77% of the true chemical bonds (17 chemical bounds out of 22 of van Raaij and co-authors). The selection provided by GeminiDistances is therefore in good agreement with van Raaij and co-authors' work.

### Chemical validation-5

The assembly process of the heptameric co-chaperone 10 from *Homo sapiens* mitochondria (*hm*cpn10) has been well studied experimentally [21], [30], [53], [54]. A particularly relevant study for us uses site directed mutagenesis to identify, among amino acids located at the interface, those which are involved in controlling the capacity of the protein to assemble into a heptamer [21]. Two residues of the interface have been mutated into glycine, Val 100 (C-terminal domain) and Phe 8 (N-terminal domain), but only the latter abolishes the formation of the heptamer. The Val 100 mutant still heptamerized but into a species less stable than the native protein. Based on this result, we would expect the Phe 8 and the Val 100 to be detected by GeminiDistances as interacting (“X”) and not interacting (“.”) amino acids, respectively. Unfortunately, there is no x-ray structure available for the whole *hm*cpn10 heptamer in the PDB allowing this straightforward test.

The sequences of both the N- and the C-terminal domains of the *hm*cpn10, domains which form the interface, have been previously compared with those of other cpn10 proteins by Guidry and co-authors [21]. The comparison is indicated in Table 6 with in addition to the previous report, the sequences of the cpn10s from *Thermus thermophilus* and from Bacteriophage T4.

**Table 6:** Protein sequence alignment of the N- and C-terminal domains of the cpn10 heptamers.

Organism	N-term	C-term	PDB code
*Human mitochondria*	K ***F^8^*** LPLFDR	DGDILGKY***V^100^***	n.a.
*Mycobacter tuberculosis*	N**I^5^**KPLEDK	ARDVLAVV**S^98^**	1HX5
*Mycobacter leprae*	K**I^5^**KPLE	ARDVLAVV**S^91^**	1LEP
*Aquifex aeolicus*	K**L^3^**RPLYDK	EDEVLAVV**E^96^**	n.a
*Thermus thermophilus*	M**I^2^**KPLGDR	LLAVL**Q^94^**	1WNR
Bacteriophage T4	P**I^9^**RAVGEY	KAIPCL**Y^110^**	1G31

The mutated residues for hmcpn10 are in italic and the corresponding ones on other cpn10s are indicated in bold. The numbers are the amino acid positions along the protein sequence. n.a stands for not applicable.

**Figure 7:**
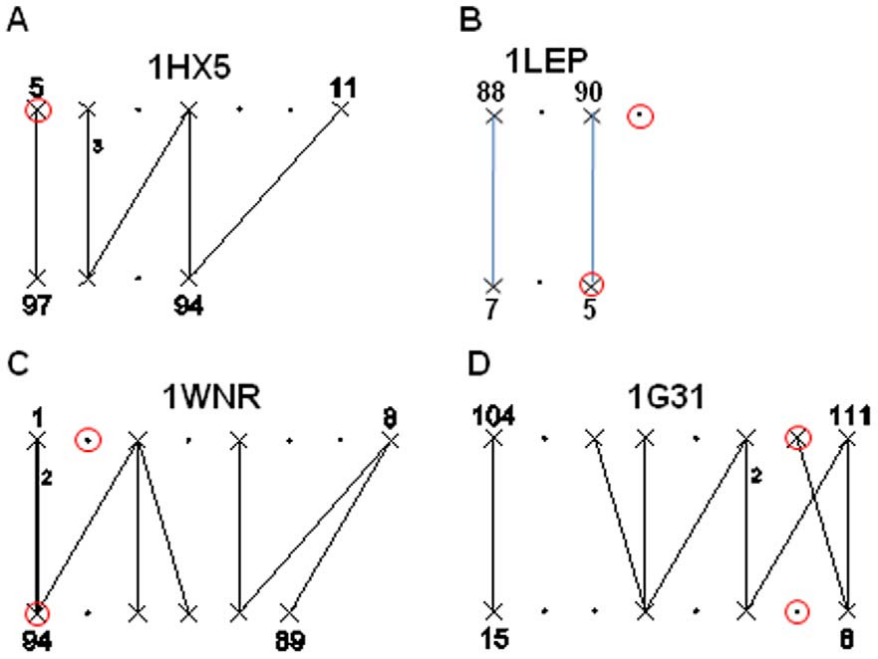
Interaction networks of the interfaces of the heptameric co-chaperon 10 for which an atomic structures is available. A. Co-chaperonin 10 from *M. tuberculosis*. B. Co-chaperonin 10 from *Mycobacter leprae*. C. Co-chaperonin 10 from *Thermus thermophilus*. D. Co-chaperonin 10 from Bacteriophage T4. The red circle indicates the residues equivalent to the amino acids mutated in the co-chaperonin 10 from *hm*cpn10 [21]. The symbols are described in Figure 3 and in methodology.

Among the cpn10s considered is the *M. tuberculosis* cpn10 (PDB name: 1HX5) which interfaces are described in Fig. 3. The region of interest is the red box one which involves interactions between residues 5 to 11 of chain A and 94 to 97 of chain G, respectively (Fig. 3C). According to the sequence alignment, the residues Ile 5 and Ser 98 of *M. tuberculosis* cpn10 correspond to Phe 8 and Val 100 of *hm*cpn10, respectively [21]. As can be seen on the interaction network of 1HX5, on the appropriate region (Fig. 3C and Fig. 7A), Ile 5 is identified as an interacting residue “X” by GeminiDistances. Thus, as expected for a proper selection by GeminiDistances, the amino acid shown to be important for the oligomerization of the cpn10, is recognized as an interacting one “X”. The residue Ser 98 is not seen on the 1HX5 x-ray structure.

We have performed a similar analysis of the interface of the three other cpn10 (from *Mycobacter leprae*, *T. thermophilus* and the Bacteriophage T4) for which a x-ray structure is available (Fig. 7 and Table 6) [55], [56], [57], [58]. The alignments and the amino acids identified as corresponding to Phe 8 and Val 100, in these three proteins, are shown in Table 6. For the *M. leprae* cpn10, similarly to what has been obtained for the *M. tuberculosis* cpn10, Ile 5 is identified as an interacting residue “X” by GeminiDistances (Fig. 7B). Ser 91 of *M. Leprae*, which corresponds to Val 100 on *hm*cpn10, is seen on the x-ray structure of the cpn0 from *M. leprae* and is identified by GeminiDistances as a non interacting residue “.” (Fig. 7B). Since Val 100 was shown experimentally to have no role in the oligomerization of hmcpn10, Geminidistances agrees again with the experimental result by detecting the Ser 91 as a “.” non interacting residue.

Now, in contrast to the previous cpn10s, the residues corresponding to the Phe 8 and the Val 100 of the *hm*cpn10 for the *T. thermophilus* and for the Bacteriophage T4 cpn10s, are identified by GeminiDistances as non interacting (“.”) and interacting (“X”), respectively, (Fig. 7C and D, respectively). Both the N- and the C-terminal residues of both *T. thermophilus* and the Bacteriophage T4 cpn10s are seen on their respective x-ray structures.

Thus, there seems to be a discrepancy between the selection of GeminiDistances and the presumed role of these amino acids in the assembly of the cpn10s, suggested by the study of the *hm*cpn10. However, the *Aquifex aeolicus* cpn10 (*Aa*cpn10), for which the *T. thermophilus* x-ray structure is used as a model, has been shown to follow a different assembly mechanism than the *hm*cpn10 [30]. Subtle differences in the amino acid compositions of the two proteins are probably responsible for their two different mechanisms of assembly [30]. It might well be that the N-terminal is important in the reassembly mechanism of the hmcpn10 but is not in the reassembly of the *Aa*cpn10 and vice-versa for the C-terminal residue. In that case, the selection performed by GeminiDistances for the cpn10s from *T. thermophilus* and from the Bacteriophage T4 would also be correct.

## Discussion

We present here a simple method to select some of the amino acids involved in the interface of a protein oligomer using its atomic structure. The method aims at providing a comprehensible picture of the 3D structure of the protein interface. The result can be subsequently used to address questions relevant to the understanding of the protein assembly. First, the computational time needs to be short such that it doesn't preclude the investigation of a large dataset, required for the comparison of many protein interfaces. Second, for this inductive theoretical analysis, the method must output the protein interfaces in a format that facilitates the identification of common structural and/or chemical features. Third, the output must be a simplified representation of the interface compared with the one provided by standard 3D visualization programs like Rasmol. Finally, the interface representation should facilitate the identification of the amino acids to be mutated to inhibit assembly.

Based on these criteria, the program called GeminiDistances was developed. It calculates distances between atoms of two neighboring chains and selects all plausible chemical interactions involved in the interface, with a cut-off distance of 5 Å (see methodology). Out of them, a subset of chemically possible interactions is retained by choosing only the closest atoms between the two adjacent chains. This procedure is called symmetrization in Methodology. The chemical properties of the atoms are not taken into account in the process. The program rapidly proposes a framework of the interactions which are chemically possible. The selected atoms belong to amino acids of the protein which are finally considered as the interacting amino acids of the interface.

GeminiGraph uses the output of GeminiDistances to visualize, by a very user-friendly 2D interaction network, some of the properties of the x-ray 3D-structure of a protein interface (see methodology). The interaction networks created by GeminiGraph are extremely useful to compare properties of many protein interfaces through simple methods.

GeminiDistances selection indicates that 20% of the amino acids of a protein oligomer are involved in the interface which is consistent with finding of other authors [8].

No preferential secondary structure is found in interfaces as the majority of them (around 60%, in all the stoichiometries from trimer to dodecamer) contains both alpha and beta secondary structures. This illustrates the diversity of geometries capable of forming interfaces. Moreover, the distribution of the different types of secondary structure (pure α, pure β and mixed) is similar throughout the stoichiometries, indicating that there is no secondary structure dedicated to a particular stoichiometry. This suggests that interfaces are made of domains, or building blocks, whose property is to stick to one another and which can be used independently from the stoichiometry. There also appear to be a general trend of more α-helical interfaces (30%) than β-sheet interfaces (10%) throughout the stoichiometries, except for the heptamers (Figure 4). This trend has also been reported by others for homodimers [8], [59]. Although to a lower extent, there are also less α-helical interfaces for pentamers and decamers than for other stoichiometries (Figure 4).

As a whole, the result of the GeminiDistances selection agrees with the literature showing a generally good assessment of the amino acids involved in protein interfaces.

Now, it has been also evaluated how much the amino acids selected by GeminiDistances characterize the geometry of a protein interface. The analysis of the interaction networks enables us to show that the amino acids selected by GeminiDistances have information related to both the secondary and the tertiary structures of the interface. To identify α or β structures, we simply need to calculate as follows: 1) the ratio of interacting amino acids to the total number of amino acids of the interface; 2) the number of amino acids separating two amino acids of the same segment, interacting with the same residue on the complementary segment. The combination of both calculations gives an accurate determination of the 2D structure of the interface.

Although the 3D-structure of the protein interface clearly conditions the interaction networks, their exact relationship remains to be established.

Moreover, the accuracy of the chemical selection made by GeminiDistances (i.e. the selection of the interacting amino acids “X”) was evaluated through the use of four different approaches. Indeed, it has been compared to the chemically involved amino acids (i) detected by hydrogen bonding computing methods, (ii) described for two well-known interfaces of α−and β−geometries (α-helix coiled coil and β-spiral interfaces, respectively), (iii) as shown experimentally and finally, (iv) detected by two other programs identifying interfacial amino acids. On average for the methods used, 87% of the amino acids identified as chemically involved in an interface have been detected as interacting ones by GeminiDistances. Moreover, 82% of the chemical bonds (i.e. two amino acids interacting with each other) identified by the three methods, have been correctly detected by GeminiDistances. More significantly, the amino acids identified experimentally as important for the assembly of the cpn10 are also properly identified as “X” by GeminiDistances.

This is important as it shows that although the GeminiDistances detections are based on the x-ray structures of the native proteins, the selected amino acids are not necessarily only involved in the maintenance of the native state but also in the formation of the interface. This supports the use of GeminiDistances to identify elements (e.g. amino acids) that regulate the assembly mechanism.

There are several other programs that perform a similar task to GeminiDistances, either by distance selection or based on solvent surface accessibility or by conservative evolution selection [12], [13], [14], [48], [49].

Using the particular case of the TolC trimer, the selections of GeminiDistances and of SCOWLP and PPIDB have been compared. It is found that 8 and 20% of the amino acids detected by GeminiDistances are false positives when compared to SCOWLP and to PPIDB, respectively. SCOWLP, which performs its selection on distances and on chemical properties of atoms, has 18% false positive when compared to PPIDB. The false negatives of GeminiDistances amount to about 16% and 39% compared to SCOWLP and to PPIDB, respectively. These high percentages of false negatives are to be expected since GeminiDistances selects only a unique interaction per atom. Thus, GeminiDistances described only a part of the interactions detected by the two other programs.

The percentages of false positives are relatively low, indicating that few amino acids are “wrongly” identified as interacting by GeminiDistances. It is important to note that GeminiDistances is in good agreement with SCOWLP whose selection has some chemical criteria. This suggests that considering the closest atoms provide a good estimate of the plausible chemical interactions. Altogether, GeminiDistances behaves reasonably well compared to these two programs: 92% and 80% of the amino acids detected as “X” by GeminiDistances are also detected by SCOWLP and by PPIDB, respectively.

As GeminiDistances, MAPPIS selects only some of the interacting amino acids of a protein interface. MAPPIS recognizes hot spot residues through conserved spatial chemical interactions [60]. On a subset of protein oligomers identified by GeminiDistances as having the same geometry of interface, some of the interacting “X” residues are also detected as hot spot residues by MAPPIS (manuscript in preparation). Interestingly, the considered proteins do not share similar function or similar global fold. Only their interfaces share the same geometry. It is therefore possible that the residues selected by GeminiDistances and by MAPPIS are conserved for a geometrical reason.

It is important to highlight that GeminiDistances, as MAPPIS, screens the 3D of protein interfaces, but in contrast to MAPPIS, GeminiDistances does not use comparison partners to perform a selection. Hence, the “hot-spot” residues can be detected in a single protein oligomer without an “a priori” knowledge of other functionally or structurally related protein oligomers.

Altogether, the study shows that GeminiDistances is accurate in detecting the amino acids geometrically and chemically involved in an interface. The chemical accuracy is particularly remarkable since the GeminiDistances selection is mainly based on geometry. This recalls Crick's concept observed on alpha-coiled coil interfaces: the analysis of the geometry of a protein interface leads to its chemical specificity [22]. The selection criteria are simple therefore the calculation time is extremely short (0.2 s per protein oligomer, in average).

The program seems robust enough to be used for addressing questions on protein assembly. Some examples of applications are briefly summarized below.

### Applications

Gemini has been made to provide information on protein interfaces that can be readily used for experimental designs.

The graph (interaction network) contains geometrical and chemical information on interfaces. Therefore it can be assumed it contains the elements necessary for the formation of interfaces. Thus, based on the graph, an experiment can be designed to test the role of the amino acids “X” in the assembly of a protein, using for exemple, site-directed mutagenesis. Graphs of different interfaces are easily compared, especially if one wants to combine sequence and 3D comparisons for choosing the “X” to be mutated.

In other words, Gemini enables fast interface analysis without having to use more elaborate 3D visualization programs or 3D comparison programs, which often require some skills.

### Public Accessibility

There is no database available yet but the Gemini outputs are provided as supplementary material (Texts S1, S2, S3, S4, S5, S6, S7, S8, S9, S10, S11). The supplementary files are named Text S1 to Text S10 for the Geminidistances outputs of trimeric to dodecameric protein oligomers. The supplementary file named “Text S11” is a manual for users. The authors are happy to provide the graphs on request. The code is protected by the French national agency for code protection.

## Supporting Information

Text S1 i03out. GeminiDistances Outputs for trimeric proteins.
(1.78 MB TXT)

Text S2 i04out. GeminiDistances Outputs for tetrameric proteins.
(4.80 MB TXT)

Text S3 i05out. GeminiDistances Outputs for pentameric proteins.
(0.25 MB TXT)

Text S4 i06out. GeminiDistances Outputs for hexameric proteins.
(0.13 MB TXT)

Text S5 i07out. GeminiDistances Outputs for heptameric proteins.
(0.07 MB TXT)

Text S6 i08out. GeminiDistances Outputs for octameric proteins.
(0.83 MB TXT)

Text S7 i09out. GeminiDistances Outputs for eneameric proteins.
(0.03 MB TXT)

Text S8 i10out. GeminiDistances Outputs for decameric proteins.
(0.16 MB TXT)

Text S9 i11out. GeminiDistances Outputs for 11meric proteins.
(0.01 MB TXT)

Text S10 i12out. GeminiDistances Outputs for dodecameric proteins.
(0.29 MB TXT)

Text S11 Readme. User's manual.
(0.00 MB TXT)
